# Potential of acylated peptides to target the influenza A virus

**DOI:** 10.3762/bjoc.11.65

**Published:** 2015-04-29

**Authors:** Daniel Lauster, Damian Pawolski, Julian Storm, Kai Ludwig, Rudolf Volkmer, Henry Memczak, Andreas Herrmann, Sumati Bhatia

**Affiliations:** 1 Humboldt-Universität zu Berlin, Institute of Biology, Invalidenstr. 42, 10115 Berlin, Germany; 2 Freie Universität Berlin, Research Center of Electron Microscopy, Fabeckstr. 36a, 14195 Berlin, Germany; 3 Charité Universitätsmedizin Berlin, Institute of Immunology, Charitéplatz 1, 10117 Berlin, Germany; 4 Fraunhofer Institute for Cell Therapy and Immunology, Am Mühlenberg 13, 14476 Potsdam, Germany; 5 Freie Universität Berlin, Institute of Chemistry and Biochemistry, Takustr. 3, 14195 Berlin, Germany

**Keywords:** amphiphilic peptide, antiviral, influenza virus, multivalency, self-assembled structures

## Abstract

For antiviral drug design, especially in the field of influenza virus research, potent multivalent inhibitors raise high expectations for combating epidemics and pandemics. Among a large variety of covalent and non-covalent scaffold systems for a multivalent display of inhibitors, we created a simple supramolecular platform to enhance the antiviral effect of our recently developed antiviral Peptide B (PeB^GF^), preventing binding of influenza virus to the host cell. By conjugating the peptide with stearic acid to create a higher-order structure with a multivalent display, we could significantly enhance the inhibitory effect against the serotypes of both human pathogenic influenza virus A/Aichi/2/1968 H3N2, and avian pathogenic A/FPV/Rostock/34 H7N1 in the hemagglutination inhibition assay. Further, the inhibitory potential of stearylated PeB^GF^ (C18-PeB^GF^) was investigated by infection inhibition assays, in which we achieved low micromolar inhibition constants against both viral strains. In addition, we compared C18-PeB^GF^ to other published amphiphilic peptide inhibitors, such as the stearylated sugar receptor mimicking peptide (Matsubara et al. 2010), and the “Entry Blocker” (EB) (Jones et al. 2006), with respect to their antiviral activity against infection by Influenza A Virus (IAV) H3N2. However, while this strategy seems at a first glance promising, the native situation is quite different from our experimental model settings. First, we found a strong potential of those peptides to form large amyloid-like supramolecular assemblies. Second, in vivo, the large excess of cell surface membranes provides an unspecific target for the stearylated peptides. We show that acylated peptides insert into the lipid phase of such membranes. Eventually, our study reveals serious limitations of this type of self-assembling IAV inhibitors*.*

## Introduction

Annually influenza A virus infections cause up to 500.000 deaths worldwide, and are therefore a serious threat, and burden to humans [1]. Hence, research and development of new affordable influenza antivirals are an important task to combat not only seasonal epidemics, but also devastating pandemics. For therapy of infected patients, several pharmaceuticals targeting influenza neuraminidase (oseltamivir, zanamivir) or the proton channel protein M2 (amantadine, rimantadine) are available. However, the efficiencies of these drugs are competing with fast and continuously changing phenotypes of the influenza virus [2].

Among different strategies to block virus entry [3], several multivalent inhibitors preventing binding of the influenza virus to the host cell proved to be potent drug candidates [4–9]. Those inhibitors bind to the virus envelope spike protein hemagglutinin (HA) which is organized as a homotrimer. In particular, inhibitors competing for the highly conserved binding site for sialic acid, which is the natural receptor presented on the host cell surface have been applied. Essentially, these approaches revealed that an efficient block of virus binding requires a multivalent interaction between virus and inhibitors. This can be rationalized by the fact that a stable binding of influenza virus to the host cell is mediated by a multivalent interaction between HA binding pockets and cell surface receptors as a monovalent interaction is too weak for stable binding [10–11].

Peptide-based self-assembled nanostructures can be used as the simplest platform for the multivalent display of ligands, although this approach has not been explored much in the context of virus inhibition. There are only a few reports on using peptide based self-assembly for influenza virus inhibition [12–14].

The entry blocker (EB) which is a peptide fragment derived from the fibroblast growth factor signal sequence 4 (FGF) has a rather broad antiviral activity among several influenza strains in the micromolar range [14]. It has been shown that EB can bind to HA, and causes viral aggregation, which has been ascribed to multimerization of EB monomers providing a multivalent surface [15–16]. However, the inhibitory mechanism has not been elucidated in detail.

Matsubara et al. introduced a sugar mimetic peptide, which binds to the sialic acid binding pocket of HA [13]. In order to increase the inhibitory capacity of the peptide, a stearyl group has been attached to the mimetic peptide, presumably leading to the formation of a supramolecular assembly, which allows multivalent interactions. By that, multivalent inhibitors could be designed with antiviral activity in the low micromolar range.

Recently, we identified an antiviral peptide, which we derived from the paratope region of an antibody directed against HA binding to the sialic acid binding pocket. The peptide was shown to bind still to this site, and inhibits different influenza A virus strains in binding, and infection being superior to other antiviral peptides. We demonstrated inhibitory performance in the micromolar range against the serotypes of human pathogenic influenza A/Aichi/2/1968 H3N2 (X31) and avian pathogenic A/FPV/Rostock/34 H7N1. Inspired by the strategy of Matsubara et al. we attached a C18 fatty acid chain to this peptide, called PeB^GF^, to assemble multivalent structures which enhanced the antiviral potential compared to the monomeric form. In this study, stearylated PeB^GF^ (C18-PeB^GF^) has been compared with EB, the stearylated sialic acid mimetic (C18-s2s), and the stearylated control peptide with the reverse amino acid sequence (C18-rs2s) in respect to their potential to inhibit virus mediated hemagglutination, and to lyse red blood cells.

## Results and Discussion

### Peptide synthesis and characterization

Peptide synthesis was performed using a rink amide resin on an automatic synthesizer by the Fmoc/*tert*-butyl strategy [17–18]. The *N*-terminus of the *N*-terminal free resin bound peptide was acylated with stearic acid using *O*-(benzotriazol-1-yl)-*N*,*N*,*N*',*N*'-tetramethyluronium tetrafluoroborate (TBTU) as coupling reagent in the presence of diisopropylethylamine (DIPEA) in DMF. Peptides summarized in Table 1 were explored for influenza A virus inhibition.

**Table 1:** Peptide sequences and modifications.

Name	Structure

C18-s2s	C_17_H_35_CO-**ARLPRTMV**-CONH_2_
C18-s2s-TAMRA	C_17_H_35_CO-**ARLPRTMV**-βA-βA-TAMRA
C18-rs2s	C_17_H_35_CO-**VMTRPLRA**-CONH_2_
C18- PeB^GFa^	C_17_H_35_CO-**XXXXXXXXXXXXXXX**-CONH_2_
Entry blocker (EB)	**RRKKAAVALLPAVLLALLAP**-CONH_2_

^a^Patent application is in progress [19]. The sequence will be revealed soon, by a publication in another journal.

Acylated and other amphiphilic peptides are well known to self-assemble into supramolecular structures [20–21]. Stearylated peptides, closely related to C18-s2s, C18-rs2s and C18-PeB^GF^ assemble as supramolecular structures with a critical micelle concentration (CMC) between 0.8–0.9 µM and a size between 0.2 and 2.3 µm depending on the peptide concentration [12]. The rather large size indicates the formation of rather large structures different from a simple sphere-like micelle. To verify and characterize the assembly of our peptides into higher-order structures, we studied exemplarily the organization of C18-PeB^GF^ by dynamic light scattering (DLS) and transmission electron microscopy (TEM).

**Figure 1:**
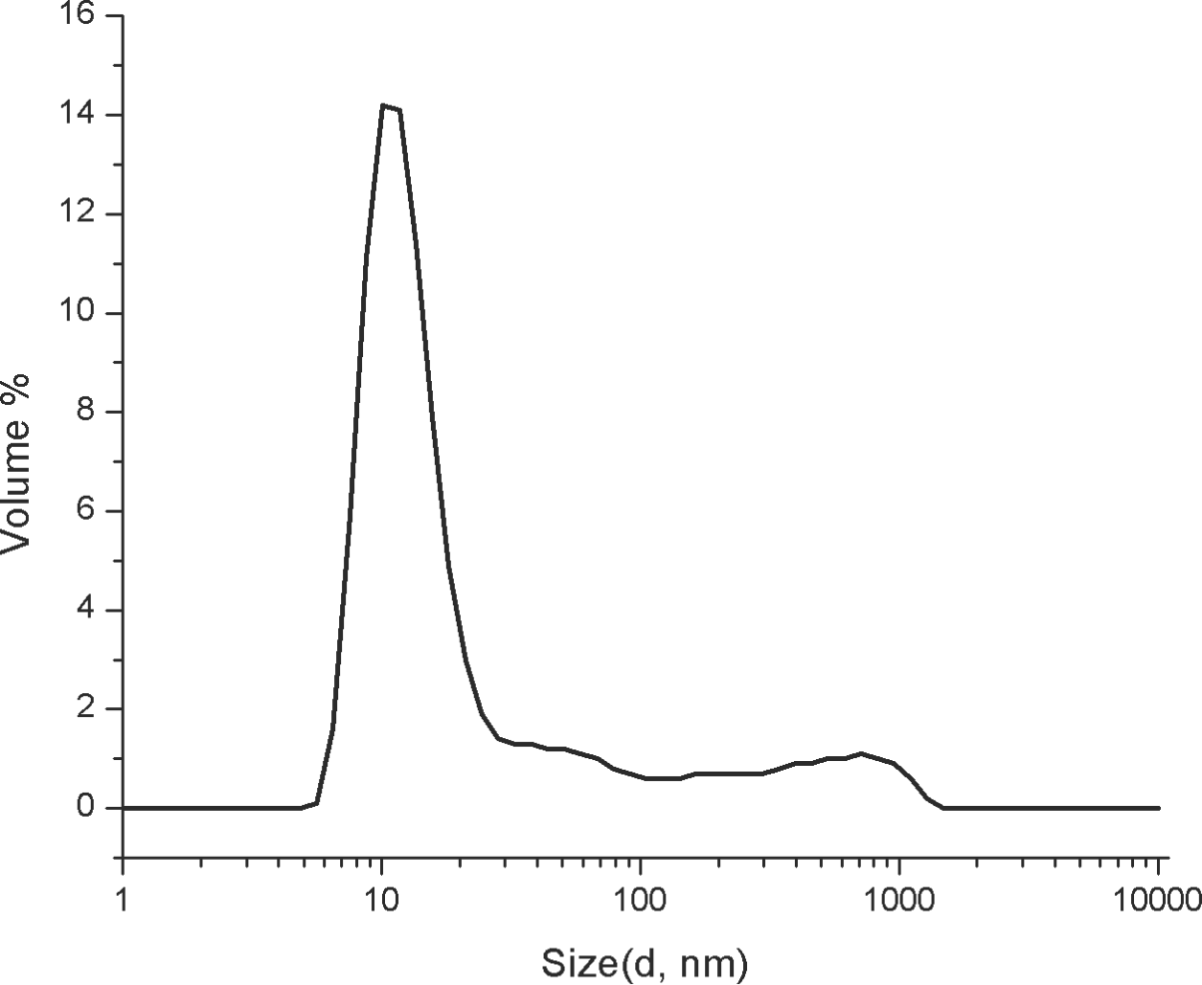
Volume size distribution profile of C18-PeB^GF^ at a concentration of 20 µM in PBS (10 mM, pH 7.4).

First, the size of the supramolecular nanostructures formed by C18-PeB^GF^, C18-s2s, and C18-rs2s was analyzed by DLS at low concentration of 20 µM in PBS (10 mM, pH 7.4). For the analysis of C18-PeB^GF^, we observed a hydrodynamic diameter of 16.7 nm (PDI = 0.454) along with 10–15% bigger supramolecular structures with hydrodynamic diameters of 184.3 nm and 573.1 nm as per volume distribution profile (Figure 1). We observed much bigger aggregates in the case of C18-rs2s with a hydrodynamic diameter of 818.8 nm (PDI = 0.328). The volume size distribution was multimodal for C18-s2s showing higher-order aggregates of different sizes at 20 µM concentration (see Supporting Information File 1).

**Figure 2:**
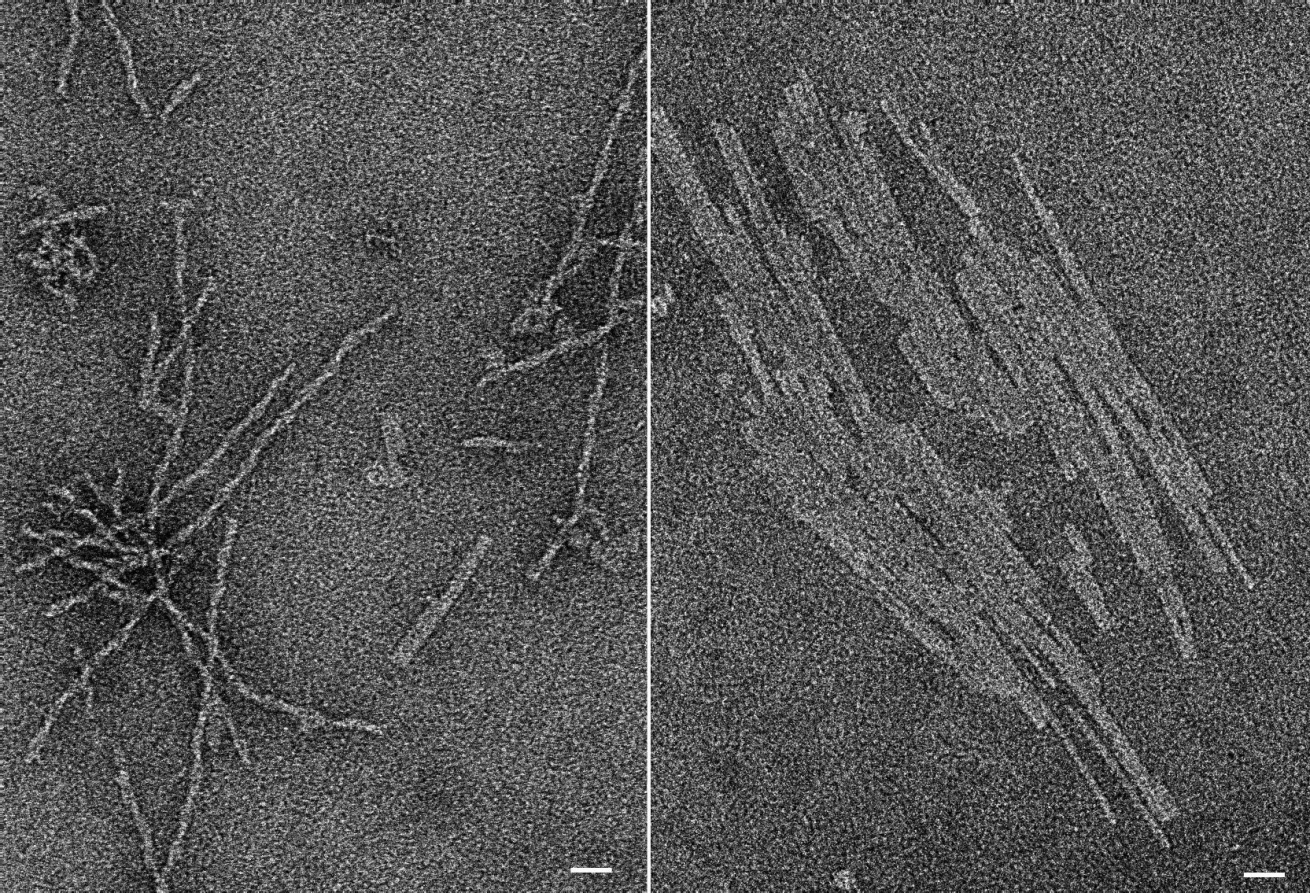
Negative staining transmission electron micrographs (TEM) of fibrillar (left) and sheet-like structures (right) of C18-PeB^GF^ (2 mM in PBS), scale bars correspond to 25 nm.

To address if the tendency of C18-PeB^GF^ to form larger supramolecular structures becomes prominent at higher concentrations, we analyzed C18-PeB^GF^ in DLS measurements at a concentration of 100 µM, too. Indeed, under those conditions we observed supramolecular aggregates with a size larger than 1 µm indicating the strong potential of C18-PeB^GF^ to organize as rather large assemblies. To visualize the organization of those assemblies, we employed TEM. To facilitate the structure identification, we used an even higher concentration of C18-PeB^GF^. We found a fibrillar supramolecular arrangement being up to several hundred nanometers long at 2 mM peptide concentrations (Figure 2). These fibers appeared predominantly as single, rather elongated structures. However, we found sheet like structures, possibly from a side-by-side assembly of these fibers, too. Importantly, cryo-TEM provides the same results (not shown) showing that formation of the large assemblies is not caused by contrast material. Preliminary TEM studies indicate similar assemblies for the other peptides used here.

Our results indicate that the amphiphilic peptides do not behave like typical micelle-forming molecules. Although we found smaller supramolecular assemblies at 20 µM C18-PeB^GF^, there is a strong tendency to form larger and stable supramolecular arrangements. Indeed, our TEM images implicate a rather amyloid-like character of C18-PeB^GF^ and other amphiphilic peptides used here. Notably, such larger structures are consistent with the observation of Matsubara et al. at least with respect to the dimension. Although the authors did not visualize the morphology of their structures, the DLS data of this report indicate different sized assembly forms even in the µm range.

### Amphiphilic peptides cause aggregation of viruses

For the stearylated peptide s2s and the polar peptide EB binding to influenza HA has been demonstrated [13–14]. In accordance with the study of Matsubara et al. we used the reverse peptide rs2s from the sialic acid mimetic as a control which does not recognize the sialic acid binding pocket and thus does not bind to HA.

**Figure 3:**
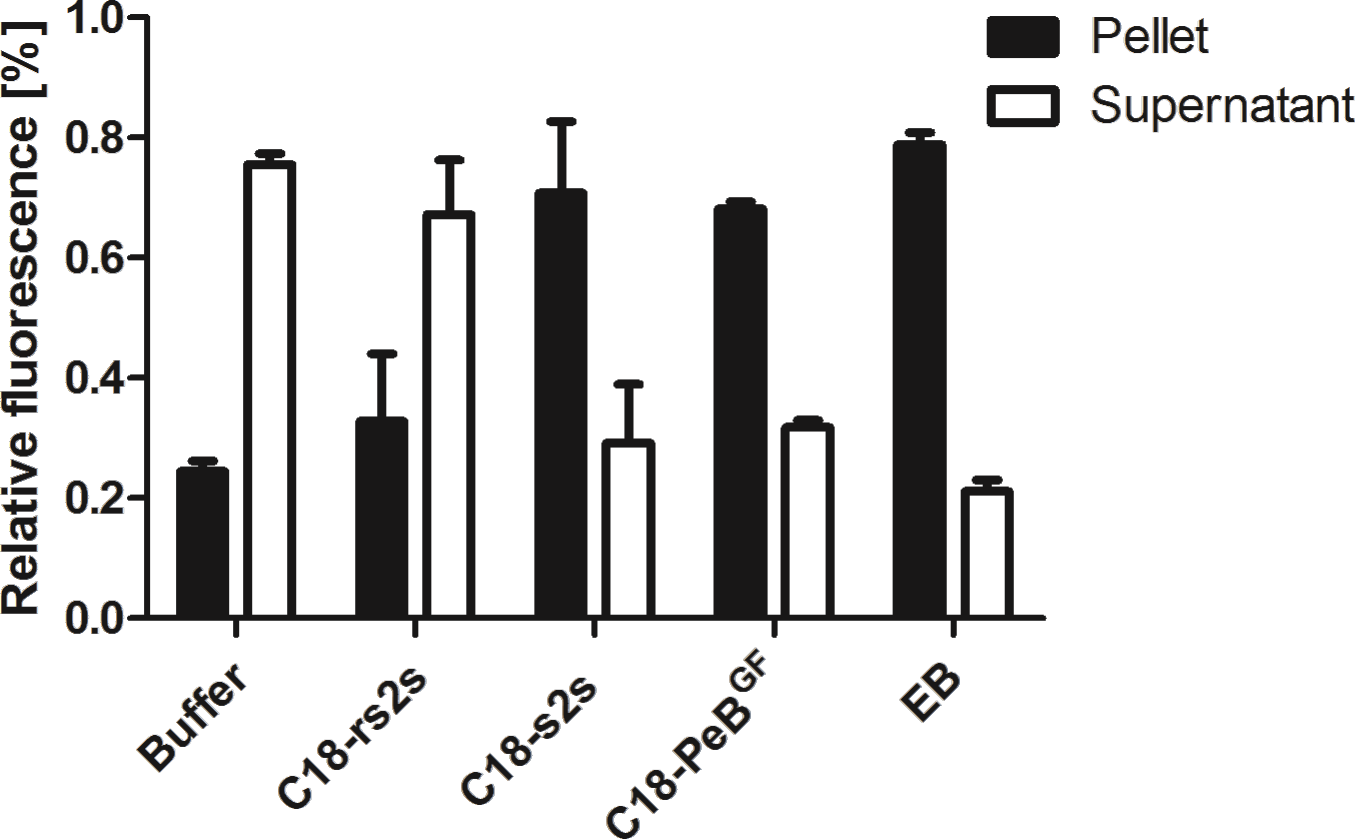
Amphiphilic inhibitors induce aggregation of viruses. R18 labeled influenza virus was incubated without or with inhibitors at 100 µM for 20 min at room temperature and subsequently centrifuged. To supernatant and pellet, respectively, Triton-X100 was added, and their fluorescence signal was recorded. Relative fluorescence indicates fluorescence from pellet and supernatant in relation to the total signal before centrifugation. Error bars represent the standard error of the mean (SEM) of at least three independent experiments.

To prove whether stearylated PeB^GF^ is able to interfere with influenza virus activity, we first investigated its potential to aggregate viruses and compared it with that of other amphiphilic peptides (Table 1). To this end, fluorescently labeled influenza A virus X31 has been incubated with amphiphilic inhibitors at 100 µM concentrations and shortly centrifuged. For all inhibitors, but the control compound C18-rs2s a higher fluorescent signal in the pellet compared to the supernatant was observed, indicating not only binding to viruses but also aggregation of viruses caused by the inhibitors (Figure 3). Jones et al. suggested that the inhibitory mechanism of action of EB is based on its viral aggregation potential, which has been supported by density gradient and electron microscopy analysis [15]. Indeed aggregation of viruses can only be explained by the formation of a supramolecular arrangement of amphiphilic peptides forming a surface with multiple ligands recognizing HA but not by a monomeric organisation of amphiphilic peptides.

### Amphiphilic peptides interfere with virus binding to cells

The potential to inhibit binding of influenza viruses to cells can be investigated by the well-established hemagglutination inhibition assay (HAI) [22]. All peptides, but the control peptide C18-rs2s were able to inhibit Aichi H3N2 virus mediated hemagglutination already at low micromolar concentrations (Table 2). For EB an IC_50_ of 20 µM against Aichi H3N2 in the HAI has been determined, however at higher viral titer than we used [14].

**Table 2:** Inhibition constants for inhibition of virus mediated hemagglutination (*K*_i_HAI) and for inhibitor caused agglutination (*K*_i_A).

Compound	C18-PeB^GF^	C18-s2s	C18-rs2s	EB

*K*_i_HAI _(Aichi H3N2)_ [µM]	1.2 ± 0.0	0.8 ± 0.5	no effect	1.6 ± 0.3
*K*_i_HAI _(Rostock H7N1)_ [µM]	2.8 ± 0.9	n.d.	n.d.	n.d.
*K*_i_A [µM]	100.0 ± 0.0	7.0 ± 0.8	4.1 ± 2.1	8.6 ± 7.0

The *K*_i_HAI represents the lowest concentration needed for full hemagglutination inhibition. The *K*_i_A value reflects the minimum concentration for agglutination caused by the inhibitor itself. The shown values represent the mean of at least three independent experiments. Extended values represent the standard error of the mean (SEM). n.d. (not determined).

However, we noted that inhibitors by themselves can trigger unspecific hemagglutination. To quantify this behaviour, we introduced another inhibitor constant (*K*_i_A), which represents the minimum concentration to cause inhibitor triggered human erythrocyte agglutination. While the multivalent organized peptidic inhibitors inhibited virus mediated agglutination already at low micromolar concentrations of about 1 µM, onset of unspecific agglutination was observed at much higher concentrations being in the range from 7 to 100 µM. It is important to note that the unspecific interaction of our compounds with cells can reduce the efficiency of compounds to prevent virus binding to cells, possibly even by incorporation into the lipid phase of membranes (see below).

**Figure 4:**
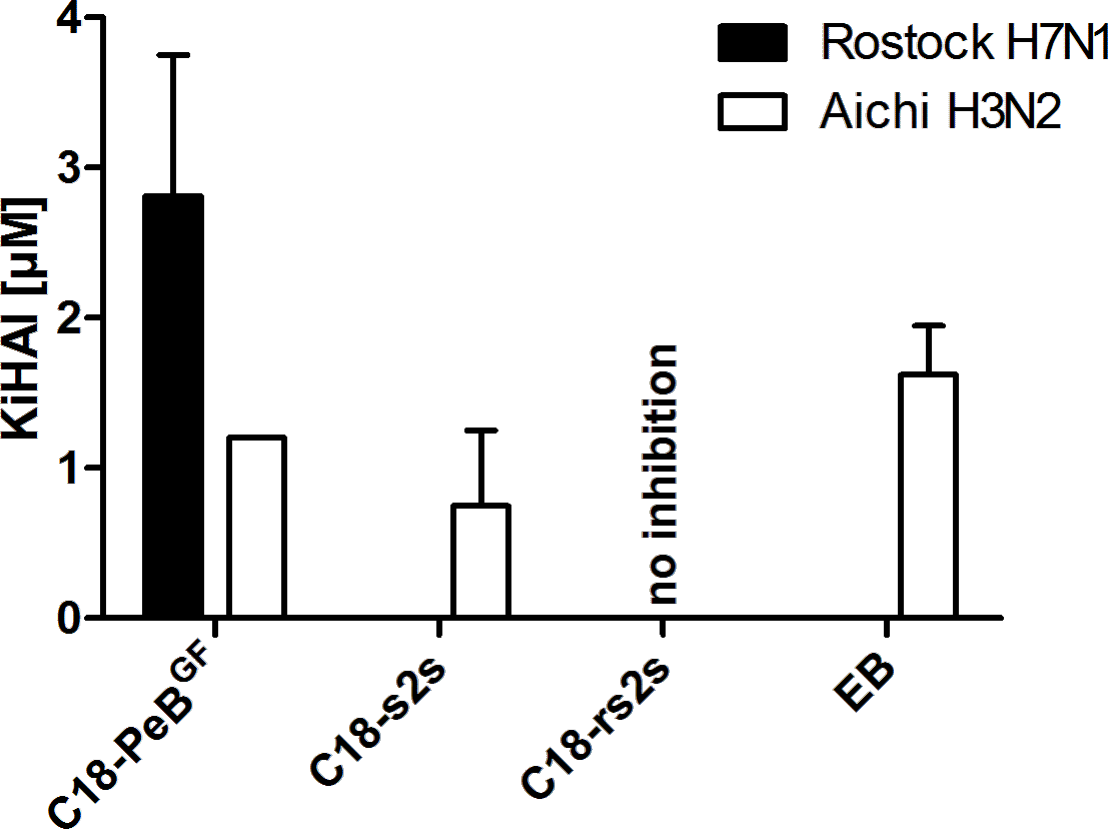
Inhibition constants *K*_i_HAI of C18-PeB^GF^, C18-s2s, C18-rs2s and EB against Aichi H3N2 and Rostock H7N1 virus. The *K*_i_HAI reflects the lowest concentration needed for full hemagglutination inhibition. Error bars indicate the standard error of the mean (SEM) of at least three independent experiments.

In addition to the human pathogenic influenza A model strain X31 (Aichi H3N2), we asked whether our inhibitor is able to inhibit hemagglutination caused by the avian pathogenic strain Rostock H7N1, too. Indeed, C18-PeB^GF^ was able to inhibit H7N1 completely at 2.8 µM concentrations (Figure 4). Thus, by attaching stearyl to the N-terminus of the PeB^GF^ sequence, we could decrease the *K*_i_HAI value up by 10 fold for H7N1 and by 20 fold for Aichi H3N2 respectively (data not shown, see patent [19]).

### Protection from virus infection by acylated peptide PeB^GF^

**Figure 5:**
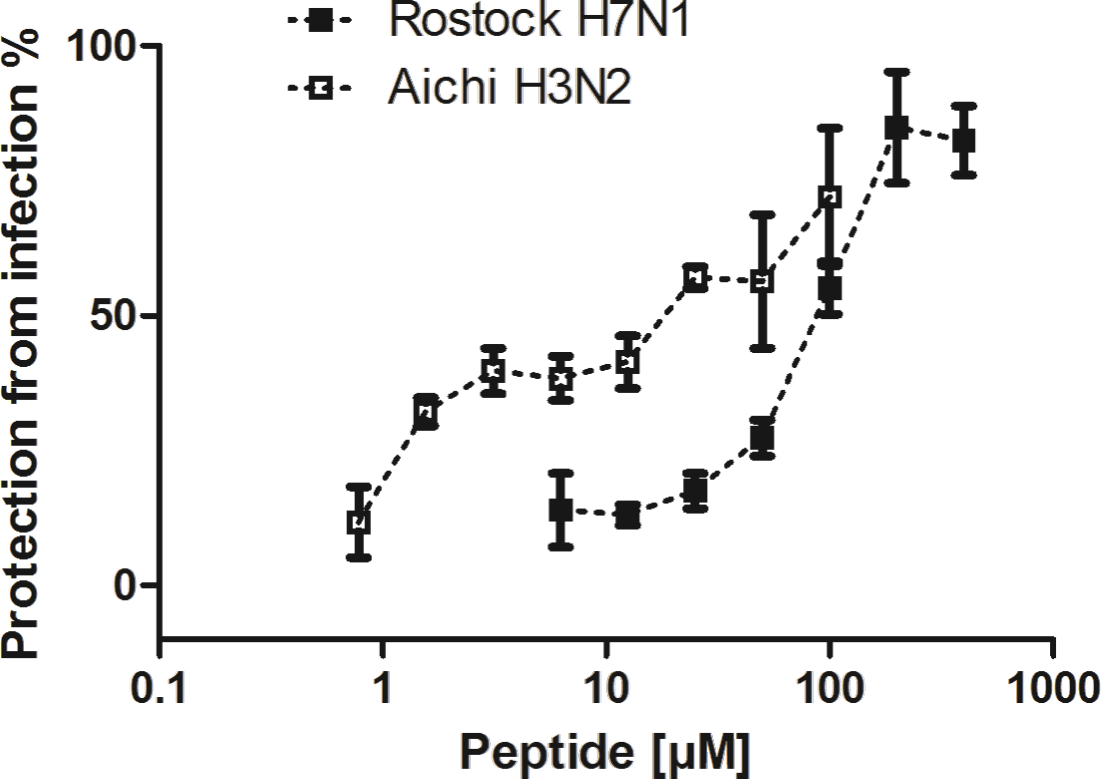
C18-PeB^GF^ mediated protection from infection of MDCK cells by Rostock H7N1 and Aichi H3N2. MDCK II cells were incubated with viruses at an MOI of 0.05 for 24 h at 37 °C. Error bars indicate the standard deviation of three experiments.

Next, we determined the potential of stearylated peptide PeB^GF^ for infection inhibition of MDCK cells by Aichi H3N2 and Rostock H7N1. We found that C18-PeB^GF^ inhibited the infection of cells at MOI 0.05 (1,500 pfu) with IC_50_ values of 84 µM against Rostock H7N1, and 5.9 µM against Aichi H3N2 (Figure 5). In comparison to unmodified PeB^GF^ the inhibitory potential could be enhanced by approx. 5 fold against Aichi H3N2, while the inhibition against Rostock H7N1 did not increase (data not shown, see patent application [19]). These results are in the same molar range found for the multivalent assemblies of C18-s2s and EB. Jones et al. determined for EB an IC_50_ of 4.5 µM against Hongkong H5N1 at an MOI of 0.05 48 h post infection.

### Interaction with lipid membranes

Although the above presented data may be promising in terms of efficient inhibition of influenza virus binding and, thus, infection, we have to take into account that the conditions of these experiments do not match the in vivo situation. Typically, such antiviral compounds will be applied intravenously or by inhalation to allow a systemic distribution or a tissue specific targeting within the infected host. However, in those cases amphiphilic peptides are in an environment of cell membrane surfaces being in excess to viruses. Thus, the majority of peptides will be incorporated into cell membranes. This would be of significant negative consequences for application of those peptides as antiviral drugs, because the multivalent presentation of the peptides would be prevented and one may speculate that cell surface membrane associated peptides may act as an additional receptor for virus attachment to the host cell surface.

Therefore, we studied the interaction of amphiphilic peptides with membranes. To this end, we repeated our hemagglutination inhibition experiments, but we incubated the peptides with 100 nm large unilamellar vesicles (LUV) containing 6.25 nmol DOPC for 30 minutes before virus and erythrocytes were added. In that case the *K*_i_HAI increased by a factor of 2–4 (data not shown; notably, a similar increase was found for *K*_i_A). This suggests that the potential of inhibitors to prevent hemagglutination must have been partially neutralized by the liposomes, either by attachment and/or incorporation into the lipid bilayer. Very likely, in case of stearylated peptides, we surmise incorporation into the bilayer via the fatty acyl chain.

**Figure 6:**
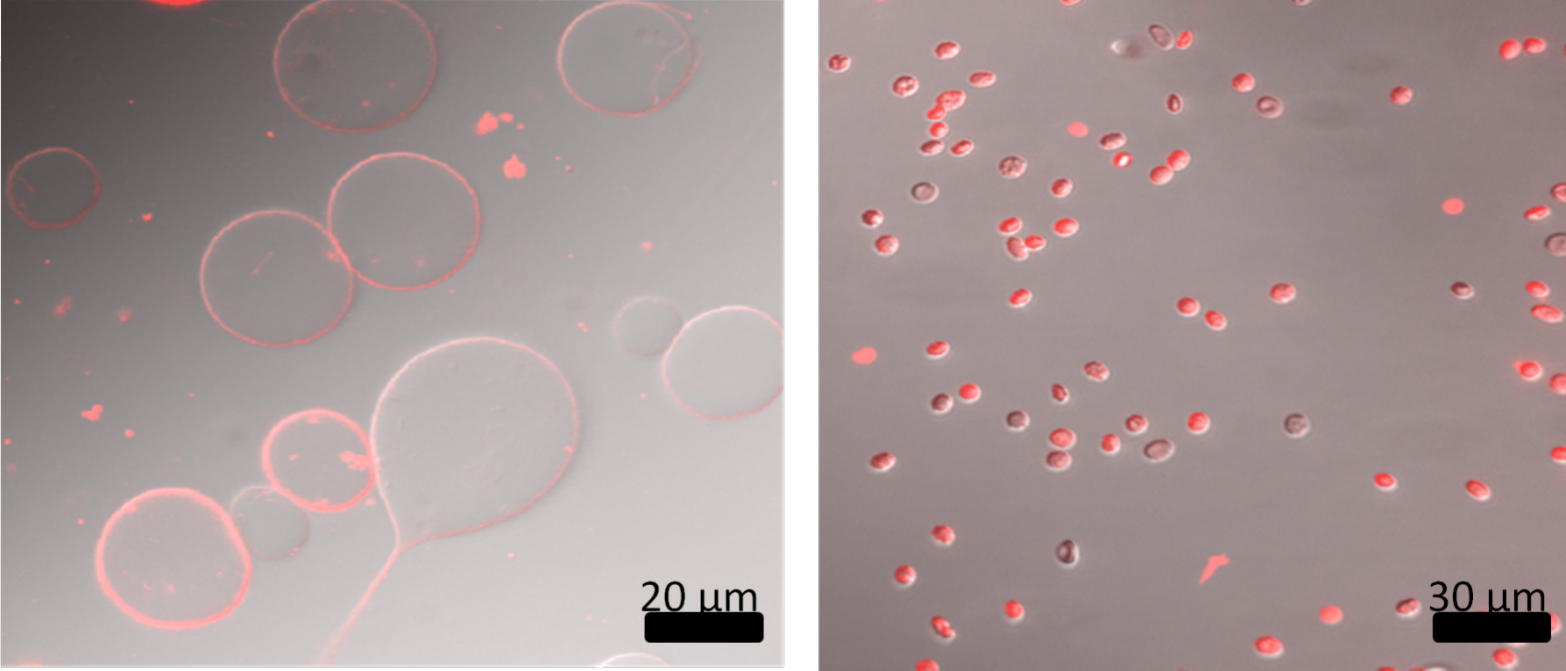
Fluorescence microscopy images of GUVs (left) and human erythrocytes (right) after incubation with C18-s2s-TAMRA. The overlap of DIC and rhodamine channels demonstrate the labeling of membranes by the fluorescent stearate peptide. Scale bars are in black.

To verify the association with lipid membranes exemplarily, we synthesized the s2s construct with a terminal rhodamine fluorophore. This compound was mixed with DOPC giant unilamellar vesicles (GUV) or human erythrocytes. In both cases clear membrane labeling could be detected (Figure 6).

**Figure 7:**
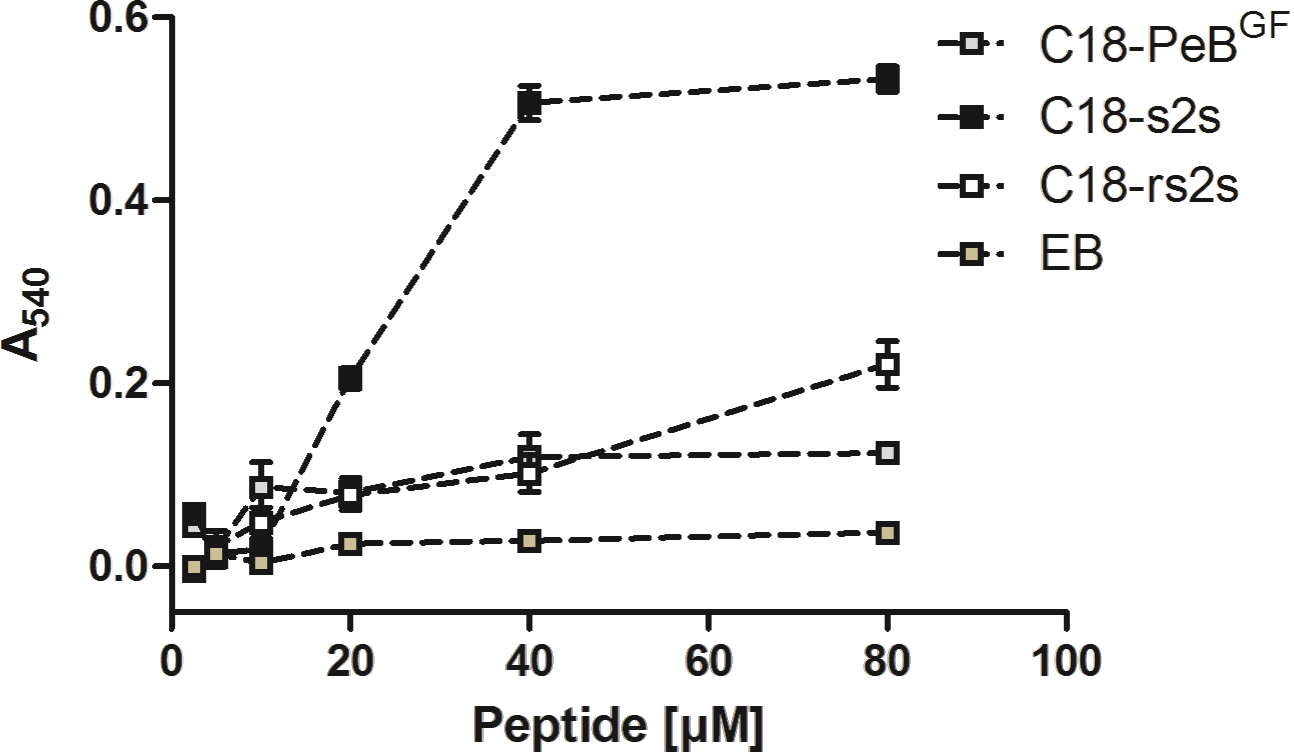
Hemolytic activity of stearylated peptides and EB. 2% human erythrocytes were incubated for one hour with peptides at 37 °C. After centrifugation, the hemolytic activity was assessed by absorption measurement of the supernatant at 540 nm. Error bars indicate the SEM of three titration experiments.

To assess how strong membrane incorporation of these peptides can perturb membranes we measured their cell lytic activity in a titration experiment with human erythrocytes. Apart from compound C18-s2s which acted extremely hemolytic above concentrations of 20 µM, all peptides showed only low hemolytic activity (Figure 7). For EB the same has been reported by Jones et al. [16].

These results show that acylated peptides, e.g., C18-PeB^GF^, could readily insert into biological membranes. As we observed association with the plasma membrane of red blood cells we surmise that those peptides could also insert into the virus envelope.

## Conclusion

Here, we investigated the potential of a stearylated HA antibody derived peptide, entitled C18-PeB^GF^ to inhibit virus binding to red blood cells, and to prevent from viral infection of MDCK II cells. Based on the known capability of amphiphilic peptides to organize as supramolecular structures, we intended to enable a multivalent presentation of virus binding ligands with enhanced antiviral activity. Although DLS analysis indicated the presence of nanostructures at lower concentrations, as the majority of detected objects showed an average diameter of 16.7 nm, we found already at 20 µM concentrations the formation of rather large supramolecular structures which are even more prominent at higher concentrations. Structural analysis by TEM revealed the presence of stable fiberlike assemblies, which can further arrange side-by-side as sheets. Thus, acylated peptides as C18-PeB^GF^ cannot be considered as micelle forming molecules as it would be expected from the C18 chain. We surmise that the peptide is an important structural determinant leading to a rather amyloid-like assembly. This is certainly a serious disadvantage for the application of those acylated peptides as antiviral drugs.

Nevertheless, only the multiple presentations of antiviral peptides, and the binding of peptides to HA can explain the observed ability to aggregate viruses, which has been demonstrated for all peptides except for the control peptide C18-rs2s. We found that C18-PeB^GF^ was able to inhibit Aichi H3N2 and Rostock H7N1 virus induced hemagglutination at 1.2 µM and 2.8 µM, respectively. In comparison to unmodified PeB^GF^ the inhibitory potential was increased by 10 fold for Rostock H7N1 and by 20 fold for Aichi H3N2. In addition, we found enhanced infection inhibition of C18-PeB^GF^ compared to its non-acylated form. Against Aichi H3N2 and Rostock H7N1 we determined IC_50_ values of 5.9 µM and 84 µM, respectively. Compared to the monomeric peptide we could reduce the IC_50_ value by 5 fold against Aichi H3N2, whereas we did not observe enhanced inhibitory potential in the infection experiments with Rostock H7N1.

However, despite the principal potential of acylated antiviral peptides such as C18-PeB^GF^ to inhibit virus interaction with cells, our observation of a strong affinity of those structures to membranes, and also to incorporate into membranes are serious disadvantages for their application as therapeutics. Indeed, we found that the inhibition of virus mediated hemagglutination by acylated antiviral peptides was drastically reduced in the presence of additional membranes (here liposomes). Taken into consideration that the in vivo situation is characterized by an excess of cell membrane surfaces serving as targets for the amphiphilic peptides, the multivalent presentation of antiviral peptides by respective nanostructures will be perturbed or even inhibited.

In conclusion, the acylation of those peptides as used in our study, and in previous studies does not resemble an advantage over other strategies of multivalent presentations.

## Supporting Information

File 1 Experimental part.
