# Supplementary material for: Potential of acylated peptides to target the influenza A virus
Source: Beilstein J Org Chem. 2015 Apr 29;11:589–95. doi: 10.3762/bjoc.11.65 (PMC4464269; doi:10.3762/bjoc.11.65)
Supplement: File 1 — Experimental part. [file Beilstein_J_Org_Chem-11-589-s001.pdf]

Supporting Information

for

Potential of acylated peptides to target the influenza A virus

Daniel Lauster[1]*, Damian Pawolski[1], Julian Storm[1], Kai Ludwig[2], Rudolf Volkmer[3], Henry Memczak[4], Andreas Herrmann[1] and Sumati Bhatia[5]*

Address: [1]Humboldt-Universität zu Berlin, Institute of Biology, Invalidenstr. 42, 10115 Berlin, Germany, [2]Freie Universität Berlin, Research Center of Electron Microscopy, Fabeckstr. 36a, 14195 Berlin, Germany, [3]Charité Universitätsmedizin Berlin, Institute of Immunology, Charitéplatz 1, 10117 Berlin, Germany, [4]Fraunhofer Institute for Cell Therapy and Immunology, Am Mühlenberg 13, 14476 Potsdam, Germany and [5]Freie Universität Berlin, Institute of Chemistry and Biochemistry, Takustr. 3, 14195 Berlin, Germany

*Corresponding author

Email: Daniel Lauster - daniel.lauster@hu-berlin.de, Sumati Bhatia - sumatichem@gmail.com

Experimental part

Methods and materials

Synthesis of unmodified and *N*-terminally modified peptides with stearic acid

All reagents and solvents were purchased from commercial suppliers and used without further purification. Peptide synthesis was performed in polypropylene syringes equipped with frits on an automatic synthesizer (Syro, MultiSynTech, Witten, Germany) applying the Fmoc-*t*-Bu-strategy. All the manufactured peptides were carrying a *C*-terminal carboxylic amide, realized by attaching the first *C*-terminal amino acid to an Fmoc-Rink amide resin (TentaGelSRam, Rapp Polymere GmbH, Tübingen, Germany). A scheduled quantity of 50 µM (ca. 200 mg resin) was applied. After Fmoc deprotection with 20% piperidine in DMF for 20 min the first Fmoc amino acid was coupled to the resin using 4 equiv Fmoc amino acid, 4 equiv PyBOP and 8 equiv NMM in DMF (30 min, repeated once). Then, the synthesis of the peptide proceeded via a repeated sequence of Fmoc deprotection and coupling of the correspondent Fmoc amino acid (Fmoc deprotection and coupling conditions see above; reaction time was extended with growing peptide length up to 1 h). For the introduction of the *N*-terminal stearic group, the *N*-terminal free resin-bound peptide was treated with 5 equiv stearic acid, 5 equiv TBTU and 10 equiv DIPEA in DMF overnight. Peptides were deprotected and cleaved from the resin using ca. 2 mL of a cleavage cocktail for each syringe (cleavage cocktail: 10 mL TFA, 0.75 g phenol, 0.5 mL water, 0.5 mL methyl phenyl sulfide, and 0.25 mL 1,2-ethandithiol). After 3 h at room temperature, the cleavage cocktail was separated; the peptide was precipitated by adding ice cold methyl *tert*-butyl ether (MTBE), and isolated by centrifugation and decanting off the remaining liquid. The pellet was washed by suspending in diethyl ether and again centrifuged and decanted (repeated up to five times).

HPLC purification and analysis were achieved using a linear solvent gradient (A: 0.05% TFA in water; B: 0.05% TFA in acetonitrile; gradient: 5–60% B over 30 min; UV detector at 214 nm; RP-18 column). The identities of the peptides were validated by mass spectrometry

using MALDI-TOF (microflex LT, BrukerDaltonik, Bremen, Germany) and ESI (Q-TOFmicro, Micromass, Manchester, UK).

MS (MALDI–TOF) calculated for $[M]_+$ EB: cal = 2083.79, found = 2082.42; $[M]^+$ C18-s2s: cal = 1209.84, found = 1208.61; $[M]^+$ C18-rs2s: cal = 1209.84, found = 1209.22; $[M]^+$ C18-PeB^{GF}: cal = 1880.89, found = fragment ions: 1073.48, 1081.99.

MS (MALDI–TOF) of EB

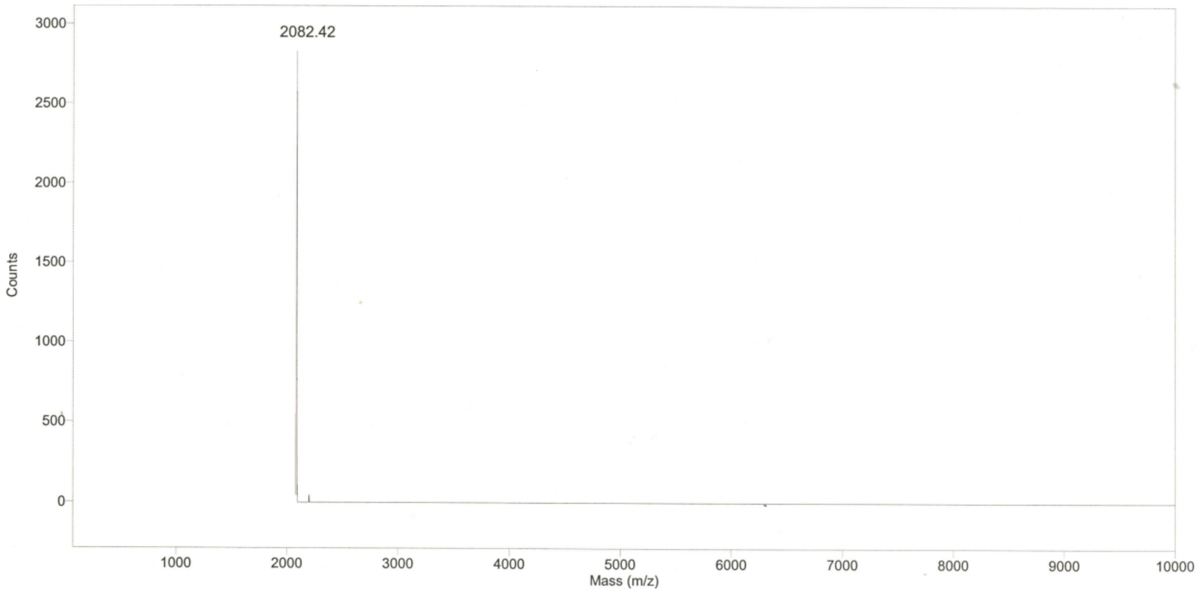

MS (MALDI–TOF) of C18-s2s

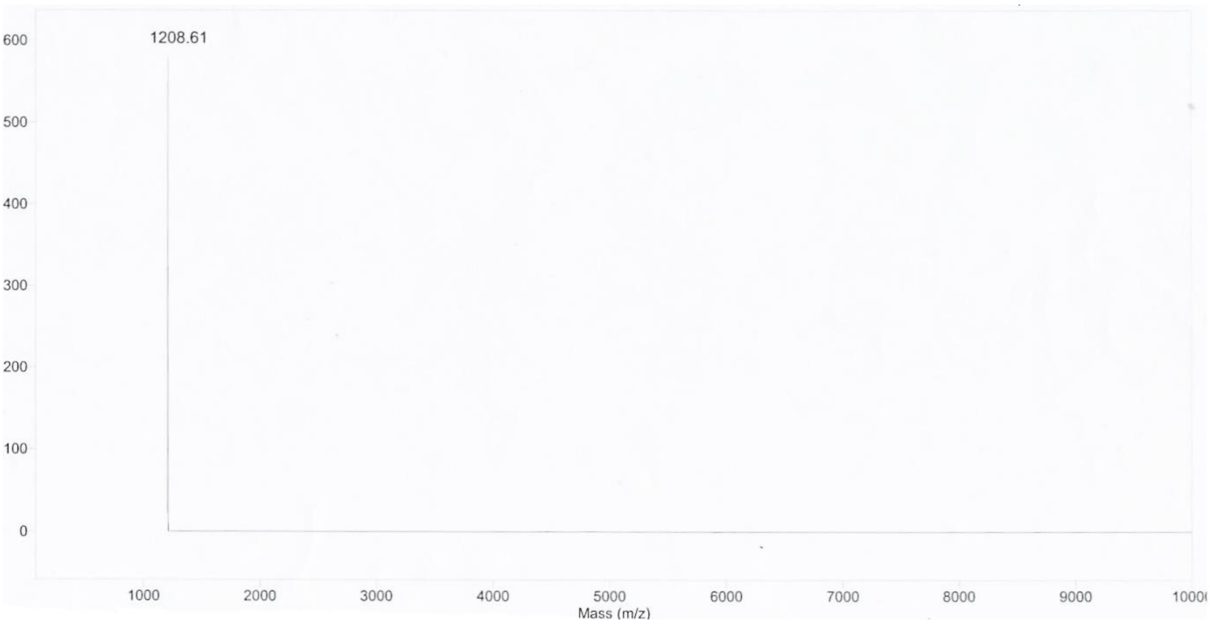

MS (MALDI–TOF) of C18-rs2s

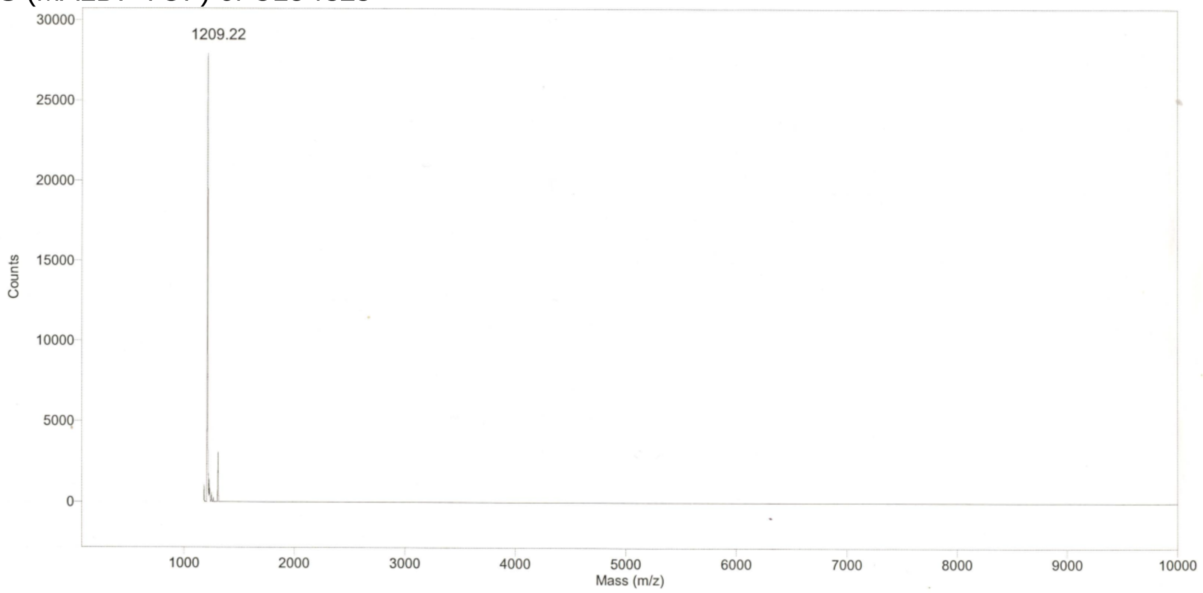

MS (MALDI–TOF) of C18-PeB[GF]

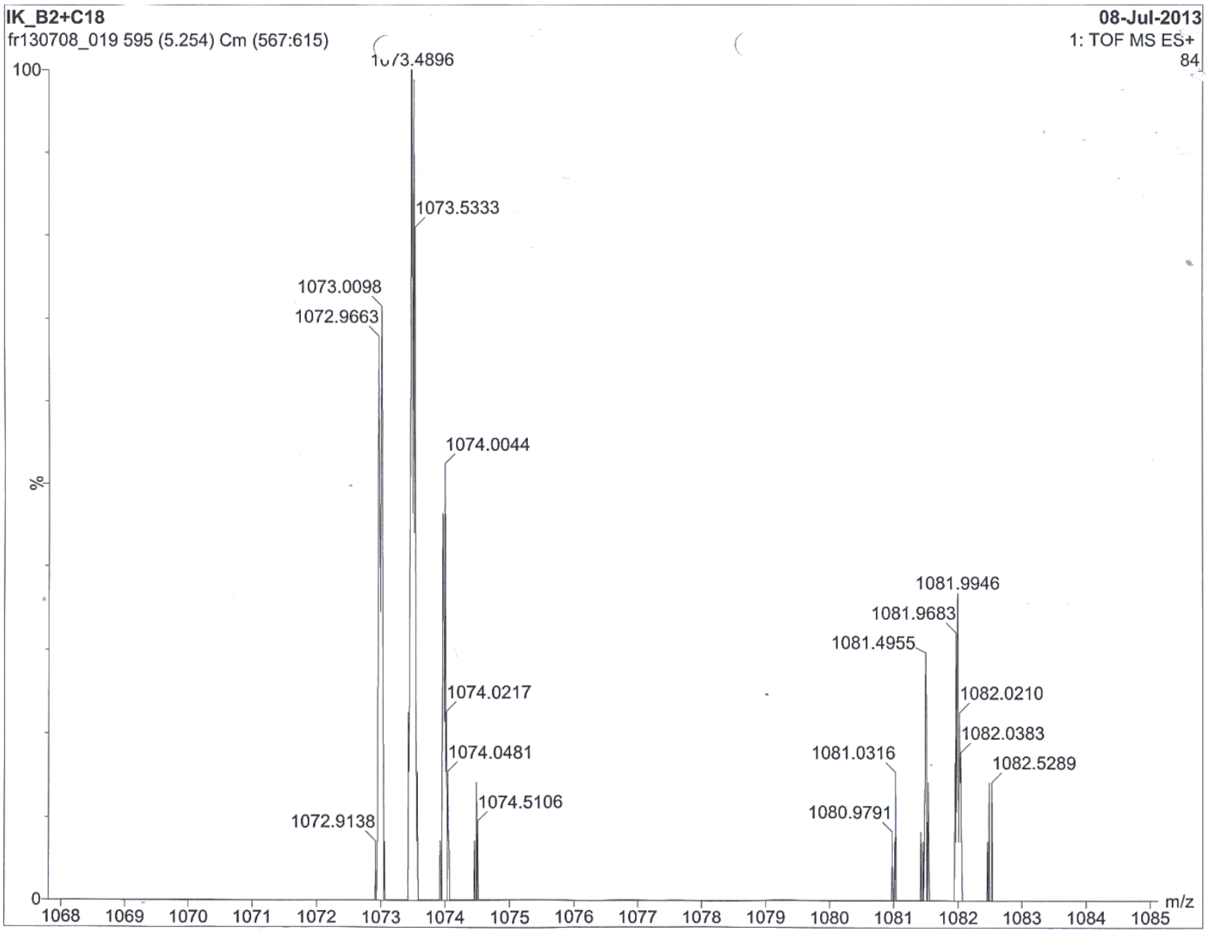

Electron microscopy

5 µL of the sample were pipetted to a hydrophilised (by 60 s glow discharging at 8 W in a BALTEC MED 020 device) carbon covered microscopical copper grid (400 mesh). After 30 s a piece of filter paper was used to adsorb excess fluid. Subsequently, 5 µL of 1% Phospho-Tungstic-Acid (pH 7.4) were applied and blotted again after 45 s. After drying in air for at least 20 min a standard holder was used to transfer the sample into a Tecnai F20 TEM (FEI Company, Oregon) equipped with field emission gun and operating at 160 kV. Micrographs were taken with an FEI Eagle 4k × 4k CCD camera.

Cryo-TEM

Sample droplets were applied to hydrophilised perforated (1 µm hole diameter) carbon film-covered 200 mesh grids (R1/4 batch of Quantifoil, MicroTools GmbH, Jena, Germany). The supernatant fluid was removed with a piece of filter paper until an ultrathin layer of the sample solution was obtained spanning the holes of the carbon film. The samples were immediately vitrified by propelling the grids into liquid ethane using a guillotine-like plunging device. The vitrified samples were subsequently transferred under liquid nitrogen into a Tecnai F20 TEM (FEI Company, Oregon) by the use of a Gatan tomography cryo-holder (Model 914). Microscopy was carried out at a 94 K sample temperature using the low-dose protocol of the microscope.

Dynamic light scattering (DLS)

Dynamic light scattering measurements were performed to determine the precise size of the supramolecular nanostructures formed by different stearylated peptides in PBS (10 mM, pH 7.4) at the concentration of 20 µM. Measurements were carried out on a Zetasizer Nano ZS analyzer (Malvern Instruments, Worcestershire WR14 1XZ, U.K.). This instrument, which uses the back scattering detection (scattering angle θ = 173°), and an avalanche photodiode

detector, is equipped with a helium-neon laser source (operating wavelength 633 nm; power 4.0 mW) and a thermostated sample chamber controlled by a thermoelectric peltier element.

DLS plots

C18-PeB^{GF}

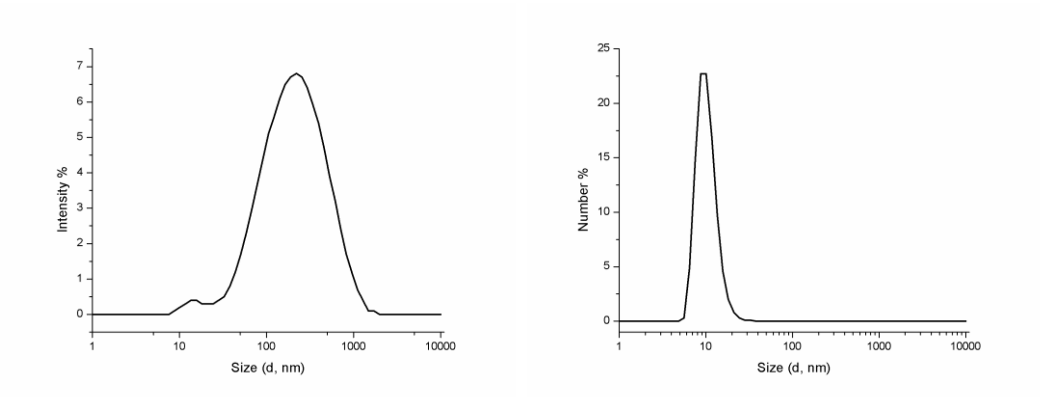

C18-s2s

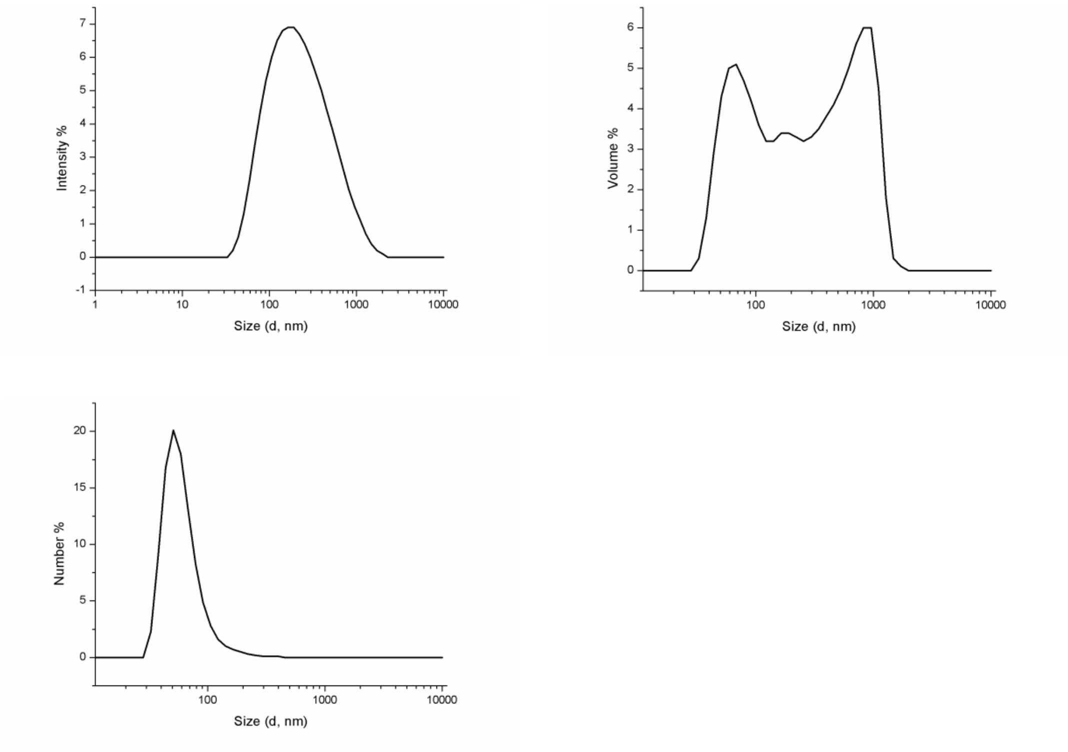

C18-rs2s

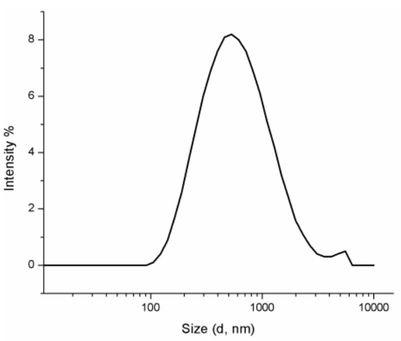

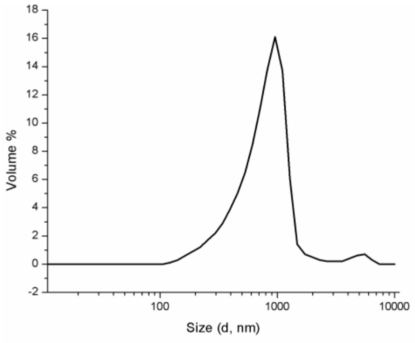

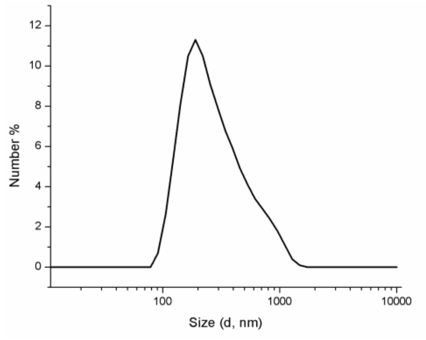

Biological material

Virus material

X31 virus (influenza strain A/Aichi/2/68 H3N2, reassorted with influenza strain A/PuertoRico/8/1934 H1N1), and low pathogenic (LP) H7N1 (A/Mute swan/Germany/R901/06; K3141) were harvested from allantoic fluid of hen eggs. LP H7N1 was kindly provided by Prof. Harder (Friedrich-Loeffler-Institut, Riems, Germany). Virus isolates were clarified upon low speed centrifugation (300 x g, 10 min) and concentrated by ultracentrifugation (100,000 x g, 1 h). Protein concentrations were determined using a standard BCA assay (Kit from Thermo Fisher Scientific, Belgium). Viral titer was determined by Tissue Culture Infectious Dose 50 ($TCID_{50}$) or hemagglutination titrations. Hemagglutination units (HAU) have been assessed with 1% human red blood cells (DRK Berlin (AB+)) in Phosphate Buffered Saline (PBS).

Cell culture

Madin-Darby Canine Kidney Epithelial II (MDCK-II) cells have been used for titration and infection experiments. The cells were cultivated in high-glucose Dulbecco's Modified Eagle Medium (DMEM), supplemented with 10% fetal calf serum, 5 mM glutamine and 1% PenStrep (100 U/mL Penicillin and 100 µg/mL Streptomycin) at 37 °C and 5% CO_2.

Experimental section

Viral aggregation assay

5 µg X31 (Aichi H3N2) virus (~160 HAU) labeled at 40 µM octadecylrhodamine B chloride (r18) was mixed with 100 µM inhibitor, and incubated for 20 min at room temperature. Then, the mixture was filled to 1 mL. Following, the remaining mixture was centrifuged for 2 min at 1,000 x g. Fluorescence values were obtained from equal volumes of supernatant and resuspended pellet, after membrane lysis with 20% (v/v) Triton X-100. After background subtraction relative fluorescence between pellet or supernatant to the initial fluorescence signal before centrifugation was calculated as following:

$$F\ (\%) = \frac{F_{pellet\ or\ supernatant}}{F_{total}}\ x\ 100$$

Large unilamellar vesicle (LUV) preparation

An 1 mM 1,2-dioleoyl-*sn*-glycero-3-phosphocholine (DOPC) solution in chloroform was filled in a glass tube and evaporated. Then, the lipid film was dissolved in PBS upon vortexing, and brought again to 1 mM. Next, multilamellar vesicles were removed in several freeze-thaw cycles (5 min at 50 °C and quickly frozen), and finally the solution was extruded ten times at 50 °C, to get the desired LUV size of 100 nm.

Giant unilamellar vesicle (GUV) preparation

A 100 nM DOPC solution in chloroform was added dropwise to a 70 °C preheated titanium plate until the plate was fully covered. A second titanium plate was sealed with the first one to form a chamber. In order to completely remove chloroform, the chamber maintained in vacuum for 1 h. Afterwards 1 mL swelling buffer (250 mM sucrose, KH_2PO_4 5.8 mM, K_2HPO_4 5.8 mM, pH 7.4 280 mosmol/kg), preheated to 55 °C, was filled into the chambers. After sealing with rubber, the chamber was connected to a voltage source (10 Hz, 20 mV–1.1 V, one step each 6 min, 2–3 h) and maintained at 50–60 °C. Following, voltage settings were changed (1.3 V and 4 Hz), and further incubated for 30 min. Finally, GUV could be taken from the chamber and stored for several days at 4 °C. For microscopy, GUV were mixed with a hypotonic microscopy buffer (250 mM D-glucose, KH_2PO_4 5.8 mM, K_2HPO_4 5.8 mM, pH 7.4, 300 mosmol/kg) in an 1:1 ratio.

Hemagglutination inhibition assay (HAI)

Inhibitors were two-fold serially diluted in PBS. Then, 2 HAU containing ~$2 \cdot 10^7$ virus particles were added to all wells. After 30 min incubation at room temperature, 50 µL of a 1% human erythrocyte solution (~$2 \cdot 10^6$ cells/µL) was added, gently mixed and incubated for another hour. The inhibitor constant K_iHAI, reflects the lowest inhibitor concentration, which is needed to achieve full inhibition. In experiments with influenza H7N1, turkey erythrocytes (kindly provided by Michael Veit, Freie Universität Berlin) were used.

Fluorescence microscopy

Confocal microscopy was conducted utilizing an inverted Olympus IX-81 microscope equipped with a cooled monochrome CCD-camera. Images were obtained with a 40× UPLAPO water objective (NA 0.95) using a 559 nm laser and a filter set (BP510-550, BA590, DM559) for red fluorescence, like r18. Further, sampling speed was set to 8 µs/pixel and the pinhole to 115 µm.

Hemolysis assay

Peptide inhibitors were serially diluted in PBS, mixed with a 2% human erythrocyte solution, and incubated for 1 h at 37 °C. Afterwards, the mix was centrifuged for 10 min at 1500 x g. Supernatants were measured for absorption at 540 nm representative for hemoglobin release. Buffer absorbance was subtracted from all data points.

Protection from infection assay

Viral infection inhibition assays were performed using an MTS reagent (Promega). 15,000 MDCK II cells were seeded the day before infection. X31 were pretreated with peptides in a two-fold dilution series for 30 min at room temperature under slight agitation. Cells were washed once with PBS (Mg^{2+}, Ca^{2+}), and pretreated virus (MOI 0.05) was added for 1 h at room temperature to allow binding. Unbound virus was removed by washing once with 37 °C infection medium (DMEM, 2 mM Glutamine, 0.1% FCS, 0.1% BSA, 2.5 µg TPCK, Pen/Strep), and subsequently incubated for 24 h at 37 °C. Two hours before the readout, 20 µL MTS solution (Promega) was added to each well and put back to 37 °C for the remaining time. Finally, absorbance at 490 nm was measured by a plate reader (Fluostar optima, BMG) and data were normalized to maximum viral cytotoxicity.

$$Protection\ from\ infection\ (\%) = \frac{(Infected_{treated} - Infected_{untreated})}{(Uninfected_{untreated} - Infected_{untreated})}$$
